# Urinary tract infections in women in Catalonia, Spain: a population-based observational cohort study in primary care

**DOI:** 10.3389/fphar.2025.1593910

**Published:** 2025-09-09

**Authors:** Silvia Fernández-García, Ana Moragas, Maria Giner-Soriano, Rosa Morros, Dan Ouchi, Ana García-Sangenís, Carl Llor

**Affiliations:** ^1^ Fundació Institut Universitari per a la recerca a l’Atenció Primària de Salut Jordi Gol i Gurina, Barcelona, Spain; ^2^ Universitat de Girona, Departament de Ciències Mèdiques, Girona, Spain; ^3^ Universitat Autònoma de Barcelona, Departament de Farmacologia, de Terapèutica i de Toxicologia, Bellaterra, Spain; ^4^ Institut Català de la Salut, Center d’Atenció Primària Jaume I, Tarragona, Spain; ^5^ CIBER en Enfermedades Infecciosas Instituto Carlos III, Madrid, Spain; ^6^ Universitat Rovira i Virgili, Departament de Medicina i Cirurgia, Reus, Spain; ^7^ Spanish Clinical Research Network, Clinical Research Unit Fundació Institut Universitari per a la Recerca a l’Atenció Primària de Salut Jordi Gol i Gurina, Barcelona, Spain; ^8^ Research Unit for General Practice, Department of Public Health, University of Southern Denmark, Odense, Denmark

**Keywords:** anti-bacterial agents, diagnosis, electronic health records, primary health care, urinary tract infections, women

## Abstract

**Introduction:**

Urinary tract infections (UTIs) are a frequent consultation in primary care and are usually treated with empirical antibiotics. Women suffer more from UTIs than men, and are therefore more likely to be treated with antibiotics. The aim of this study was to describe the epidemiology of UTIs in women in Catalonia, Spain.

**Methods:**

We conducted a population-based observational cohort study that included women patients diagnosed with UTI and recurrent UTI within the SIDIAP and CMBD database during the period from 2012 to 2021. UTI diagnoses were grouped into two main groups, cystitis and pyelonephritis, and patients recorded as having 2 or more episodes of UTI within the year following the first recorded UTI were considered to have recurrent UTI.

**Results:**

The study identified 962,998 women with at least one UTI episode. Cystitis was the most frequently reported infection and was primarily treated with fosfomycin, aligning with local clinical guidelines. Pyelonephritis was typically managed with penicillins. Recurrent UTIs accounted for 3.7% of the sample.

**Discussion:**

This large-scale cohort study provides a comprehensive overview of the epidemiology, management, and treatment of urinary tract infections in women in Catalonia. The findings highlight the widespread use of guideline-recommended antibiotics in the routine clinical management of cystitis, but not of pyelonephritis, and underscore the burden of recurrent infections in a small yet clinically significant subgroup of patients.

## Introduction

Urinary tract infections (UTIs) are a common infection in primary healthcare (PHC), and most cases are treated with antibiotics (Malmartel and Ghasarossian, 2016; Tandogdu and Wagenlehner, 2016; Kornfält Isberg et al., 2019). UTIs are more common in women than in men, with almost 60% of women experiencing at least one episode during their lifetime (Foxman, 2002; Fihn, 2003). Factors such as parity, history of abortion, sexual behavior, water intake, and urination habits have been identified as key contributors to the increased risk of UTIs (Mavi et al., 2024). In addition, some women will have recurrent UTIs, which are defined as 2 episodes in 6 months or 3 episodes in a year (Bonkat et al., 2024).

The management of UTIs is based on the prescription of empirical antibiotic treatment. The choice of antimicrobial agent is determined by the most frequently implicated pathogen and local resistance patterns. Currently, the treatment of choice in our setting is a single dose of fosfomycin or 5–7 days of nitrofurantoin for cystitis and 7 days of a cephalosporin for pyelonephritis. In addition, in the treatment of recurrent UTIs, non-pharmacological measures or non-antibiotic drugs are recommended (de Cueto et al., 2017; Almirante Gragera et al., 2021; Bonkat et al., 2024; Martín Zurro et al., 2024).

Several studies have suggested that up to 50% of antibiotic prescriptions are inappropriate (Gharbi et al., 2019; Shallcross et al., 2020), leading to the development of antibiotic resistance. Episodes of uncomplicated UTI are attributed to *Escherichia coli* in approximately 80% of cases, requiring an antibacterial agent that is effective against this pathogen (Palou et al., 2011; de Cueto et al., 2017; Rodriguez-Mañas, 2020). Resistance of uropathogens to common antibiotics has increased significantly in Spain in recent years (European Centre for Disease Prevention and Control, 2020). Resistance rates of *Escherichia coli* to fosfomycin and nitrofurantoin remain below 5% in Catalonia, according to current resistance data. However, resistance against amoxicillin and clavulanic acid, and quinolones, currently exceeds 20% of strains (Programa VINCat, 2025). To prevent an increase in antibiotic resistance, it is important to evaluate the empirical antibiotic treatment used in our area in clinical practice, and to assess whether it is in line with the observed resistance rates in order to identify areas for improvement.

The aim of our study was to describe the epidemiology of UTIs, as well as their management and treatment, in women in Catalonia, Spain, over a 10-year period. To achieve this, we analysed different local databases that provide information on clinical practice in PHC. This study is part of the project called “Urinary Tract Infections in Catalonia” (Infeccions del tracte urinari a Catalunya -ITUCAT-), which comprises multiple work packages (Moreno et al., 2023). The main project is registered in the HMA-EMA Catalogues of real-world data sources and studies with the code EUPAS49724 (European Medicines Agency (EMA), 2024).

## Materials and methods

This was a population-based observational cohort study. The inclusion period was from 1 January 2012, to 31 December 2021. The study population consisted of all women aged ≥18 years with a diagnosis of UTI registered in SIDIAP with at least 1 year of follow-up during the study period. UTI diagnoses were made according to International Statistical Classification of Diseases and Related Health Problems, 10th Revision, Clinical Modification (ICD-10-CM) codes and were grouped into 2 major groups: cystitis (N30, and N39) and pyelonephritis (N10).

The data needed to carry out the project were obtained from the SIDIAP database, and the Minimum Basic Data Sets ([CMBD] of Hospital Discharges and Emergency Departments) registries (Servei Català de la Salut, 2017).

The SIDIAP contains pseudonymised clinical information from the Electronic Health Records in Primary Care (Estació clínica d’atenció primària) program (Information System for Research in Primary Care, 2022), which is the electronic health record program for PHC of the Catalan Health Institute (Institut Català de la Salut [ICS]) in Catalonia. The ICS manages 328 PHC centres, covering a population of 5.8 million people (approximately 80% of the Catalan population) (Recalde et al., 2022). Information is available for more than 3,384 health professionals who care for the adult population.

The data recorded in SIDIAP contains sociodemographic data; health conditions, coded by ICD-10-CM (World Health Organization, 2016); clinical parameters; tobacco and alcohol use; diagnostic procedures; PHC laboratory test results; specialists referrals; and prescriptions of PHC medical staff, with the corresponding pharmacy invoice data, registered as anatomical, therapeutic, chemical (ATC) classification system codes (World Health Organization, 2024). Several reports have shown that SIDIAP data are useful for epidemiological research (Bolíbar et al., 2012; Recalde et al., 2022). SIDIAP is listed in the HMA-EMA Catalogues of real-world data sources and studies (European Medicines Agency (EMA), 2024).

The CMBD is a population-based registry that collects information on conditions treated in the health centers of Catalonia (Servei Català de la Salut, 2017) and includes ICD-10-CM codes (World Health Organization, 2016). This registry contains information provided by all Catalan healthcare centres on healthcare activity and morbidity. The CMBD of Hospital Discharges contains information on acute hospitalizations, with reasons and dates for hospital admission, while the CMBD of Emergency Departments reports activity in emergency departments.

### Study variables

The study variables included were sociodemographic information, including the MEDEA index (a socioeconomic deprivation score including five discrete values); clinical variables and health conditions, with ICD-10-CM codes (Supplementary Material S1; Table 1); tobacco and alcohol consumption; PHC laboratory test requests and results; prescriptions, with their corresponding pharmacy invoice data registered as ATC codes; CMBD-HA hospital information and CMBD-UR emergency department referral information.

**TABLE 1:** Sociodemographic characteristics of patients diagnosed with UTI.

Sociodemographic characteristics	Cystitis	Pyelonephritis	SMD^a^
Number of patients	822,530 (96.5%)	29,586 (3.5%)	
Age			
Age (mean (SD))^b^	52.34 (20.87)	44.52 (18.43)	0.397
≤20	36,026 (4.4)	1,720 (5.8)	0.406
(20,30]	114,660 (13.9)	6,176 (20.9)	
(30,40]	131,570 (16.0)	6,502 (22.0)	
(40,50]	126,767 (15.4)	5,406 (18.3)	
(50,60]	110,412 (13.4)	3,771 (12.7)	
(60,70]	106,384 (12.9)	2,471 (8.4)	
(70,80]	99,655 (12.1)	2,053 (6.9)	
>80	97,056 (11.8)	1,487 (5.0)	
Nursing home			
Nursing home (%)	46,908 (5.7)	657 (2.2)	
Area (%)			
Rural	127,334 (15.5)	3,587 (12.1)	0.100
Urban	565,058 (68.7)	21,377 (72.3)	
MEDEA Index (%)^c^			
Urban first quintile (least deprived)	102,487 (12.5)	3,855 (13.0)	0.080
Urban second quintile	111,072 (13.5)	4,067 (13.7)	
Urban third quintile	116,688 (14.2)	4,371 (14.8)	
Urban fourth quintile	119,120 (14.5)	4,609 (15.6)	
Urban fifth quintile (most deprived)	115,691 (14.1)	4,475 (15.1)	

^a^ Standardized Mean Difference.

^b^ In years.

^c^ MEDEA, index was categorized in five quintiles.

For this study, an episode of UTI was defined as the date of the first diagnosis of UTI within a 14 days interval, regardless of the number of diagnoses recorded during this interval. Outside this interval, a new record of a diagnosis of UTI was considered a new episode of UTI. Furthermore, in order to define the parameters for antibiotic treatment, the request for PHC laboratory tests, referrals to specialized care, or the presence of a urinary catheter were deemed part of the same period of the UTI if they had been registered the 15 days preceding the index date of the UTI and up to 1 month thereafter. For the comparison study between recurrent and non-recurrent infections, recurrent UTI was defined as a patient having 2 or more episodes of UTI in 1 year of follow-up since data index.

To describe antibiotic therapies, a categorization based on ATC groups was perfomed and some modifications were made (Supplementary Material S1; Table 2). For the beta-lactam antibacterial agents, penicillins and other beta-lactam antibacterial agents have been differentiated. Likewise, within the ATC category encompassing macrolides, lincosamides, and streptogramins, only macrolides were considered. Fosfomycin and nitrofurantoin have also been described as a group. ATC groups not shown in Table 4 were included under the ‘other antibiotics’ group. Antibiotic treatment for recurrent UTIs was also described by ATC, with the exception of nitrofurantoin, fosfomycin and trimetropim (guideline-recommended prophylactic antibiotics), which were given continuously during the period of 1 year after the data index, differentiating into two broad groups (less than 3 months, and more than 3 months).

**TABLE 2:** Baseline clinical characteristics of patients diagnosed with UTI.

Baseline clinical characteristics	Cystitis	Pyelonephritis	SMD^a^
Number of patients	822,530 (96.5%)	29,586 (3.5%)	
Smoking status (%)^b^			
Non-smoker	502,496 (66.6)	15,083 (58.2)	0.091
Former smoker	134,191 (17.8)	7,106 (27.4)	
Smoker	117,495 (15.6)	3,722 (14.4)	
Alcohol (%)^b^			
No risk	443,819 (71.5)	13,267 (68.2)	0.086
Moderate risk	173,881 (28.0)	6,040 (31.0)	
High risk	2,994 (0.5)	151 (0.8)	
Body Mass Index			
Body Mass Index (mean (SD))^b^	27.78 (5.87)	27.15 (6.19)	0.104
Body Mass Index >30 (%)	139,600 (31.5)	3,865 (28.8)	0.059
Comorbidites no related to urinary tract			
Diabetes mellitus (%)	27,499 (3.3)	842 (2.8)	0.029
Dyslipidemia (%)	46,550 (5.7)	1,149 (3.9)	0.083
Diseases of the nervous system (%)	11,761 (1.4)	224 (0.8)	0.065
Cerebrovascular diseases (%)	8,589 (1.0)	204 (0.7)	0.038
Musculoskeletal system and connective tissue diseases (%)	12,025 (1.5)	356 (1.2)	0.023
Diseases of the digestive system (%)	6,979 (0.8)	274 (0.9)	0.008
Comorbidites related to urinary tract			
Urinary lithiasis (%)	3,355 (0.4)	431 (1.5)	0.109
Chronic renal insufficiency (%)	9,794 (1.2)	301 (1.0)	0.017

^a^ Standardized Mean Difference.

^b^ Variables with missing data (8.5% in smoking status, 24.9% in alcohol, and 46.5% in body mass index).

### Statistical analysis

The index date was defined as the onset of each UTI episode, from which we retrospectively captured patient demographics, clinical characteristics, medication use, comorbidities, specialist referrals, and laboratory data using standardized data capture protocols with explicit handling of missing data. Specific temporal criteria were applied to each data element: active diagnoses, active medications, while BMI and laboratory values used the most recent measurement within a specific day window surrounding the index date. Our primary analysis included all women with ≥1 UTI episode during the study period, with descriptive statistics presented overall and stratified by UTI diagnosis type. Categorical variables were summarized as frequencies and percentages, with continuous variables reported as either mean (standard deviation) or median (interquartile range) based on distributional characteristics. Between-group balance was assessed using standardized mean differences (SMD), where we considered SMD values ≤0.1 to indicate adequate balance. The distribution of antibiotic treatments among patient groups was ordered by frequency and by single or combination treatments.

## Results

**FIGURE 1:**
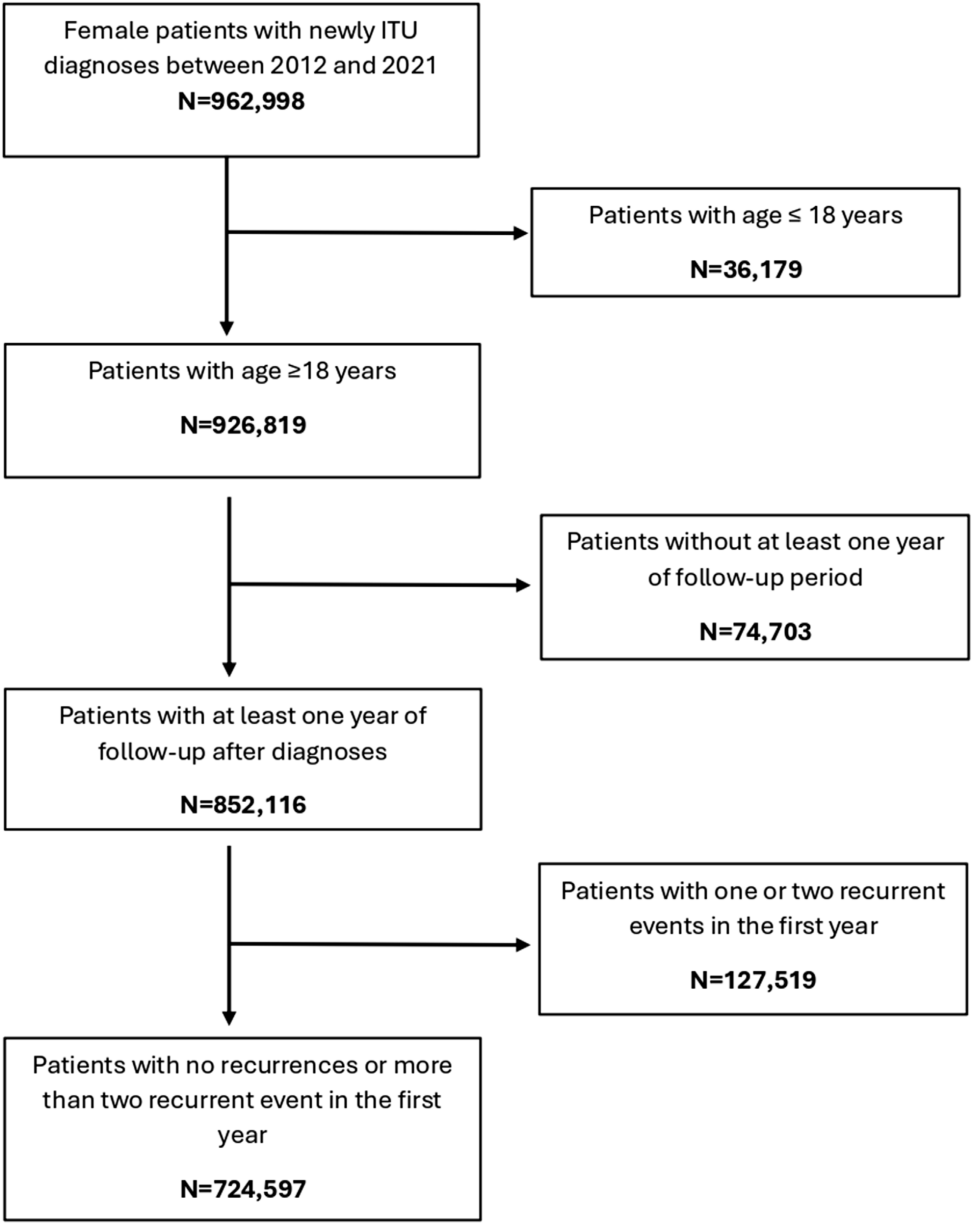
Flowchart of participants in cohort study.

Out of 2,396,441 women included in the databases between 2012 and 2021, 962,998 had a record of at least one episode of UTI. After excluding women who did not meet the inclusion criteria (Figure 1), the final sample consisted of 852,116 women.

The mean age of women with UTIs was 52 years for those diagnosed with cystitis and 45 years for those diagnosed with pyelonephritis. The highest number in both groups was observed in the 30–40 years age group and decreased with age. No differences were found across the MEDEA Index (Table 1).

Regarding lifestyle factors and comorbidities, non-smokers and non-risk alcohol consumption were found in the majority of patients. It was also observed that about 30% of the patients had a BMI above 30 (Table 2), taking into account the missing data. With regard to non-urinary tract comorbidities, dyslipidemia was the most common, followed by diabetes mellitus. In contrast, urinary tract comorbidities were relatively uncommon (less than 1.5%).

In our setting, the median follow-up from the first episode was 6 years. During this follow-up, about half of the women with UTI had at least one further event. However, when follow-up was limited to the first year after the first event, this percentage of women was reduced by more than half. In both situations, the most common event recorded during this period was a further UTI episode.

Antibiotic treatment was recorded in 82.2% and 61.2% of cases of cystitis and pyelonephritis respectively in the first episode (Table 3). The majority of women were prescribed only one course of antibiotic; fewer than 20% of patients prescribed two or more. About 30% of patients had a urine culture and a small percentage had a record of urinary catheterisation. Although hospital referral rates were low overall, referrals for pyelonephritis were higher than those for cystitis.

**TABLE 3:** Characteristics of UTI episode.

Characteristics	Cystitis	Pyelonephritis	SMD^a^
Number of patients	822,530 (96.5%)	29,586 (3.5%)	
Recurrent events and Follow-up			
Women with recurrent events (%)	407,696 (49.6)	16,535 (55.9)	0.127
1 Episode	192,048 (47.1)	7,499 (45.4)	0.042
2 Episodes	94,280 (23.1)	3,825 (23.1)	
3 Episodes	49,797 (12.2)	2,143 (13.0)	
4 Episodes	27,986 (6.9)	1,162 (7.0)	
5 Or more episodes	43,585 (10.7)	1,906 (11.5)	
Recurrent event in the first year after index date (%)	146,599 (17.8)	7,469 (25.2)	0.181
Number of recurrent events in the first year (%)			
1 Episode	121,592 (82.9)	5,927 (79.4)	0.099
2 Episodes	20,929 (14.3)	1,243 (16.6)	
3 Episodes	3,352 (2.3)	238 (3.2)	
4 Episodes	593 (0.4)	47 (0.6)	
5 Or more episodes	133 (0.1)	14 (0.2)	
Recurrent patients defined as having ≥2 episodes in the first year after index date (%)	25,007 (3.0)	1,542 (5.21)	0.135
Follow-up, in years (mean (SD))^b^	5.57 (2.74)	6.01 (2.72)	0.161
median [IQR]	5.58 [3.11, 7.93]	6.07 [3.71, 8.38]	
Treatment (%) - First episode			
No register	146,088 (17.8)	11,482 (38.8)	0.482
1 Antibiotic	555,949 (67.6)	14,601 (49.4)	
2 Antibiotics	104,509 (12.7)	3,032 (10.2)	
>2 Antibiotics	15,984 (1.9)	471 (1.6)	
Other clinical events			
Urine culture (%)	222,543 (27.1)	9,211 (31.1)	0.090
Urinary catheter (%)	1,718 (0.2)	332 (1.1)	0.113
Septicaemia (%)	1,242 (0.2)	79 (0.3)	0.025
Specialized care referrals (%)^c^	18,368 (2.2)	3,577 (12.1)	0.389

^a^ Standardized Mean Difference.

^b^ In years.

^c^ Registration of specialized care referrals from primary care.

**FIGURE 2:**
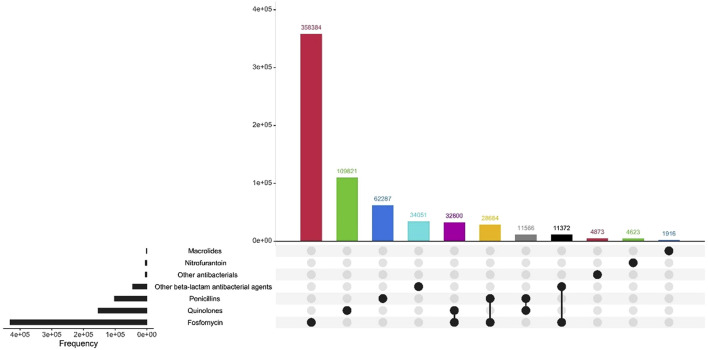
Antibiotic treatment of patients diagnosed with UTI, by treatment group.

**FIGURE 3:**
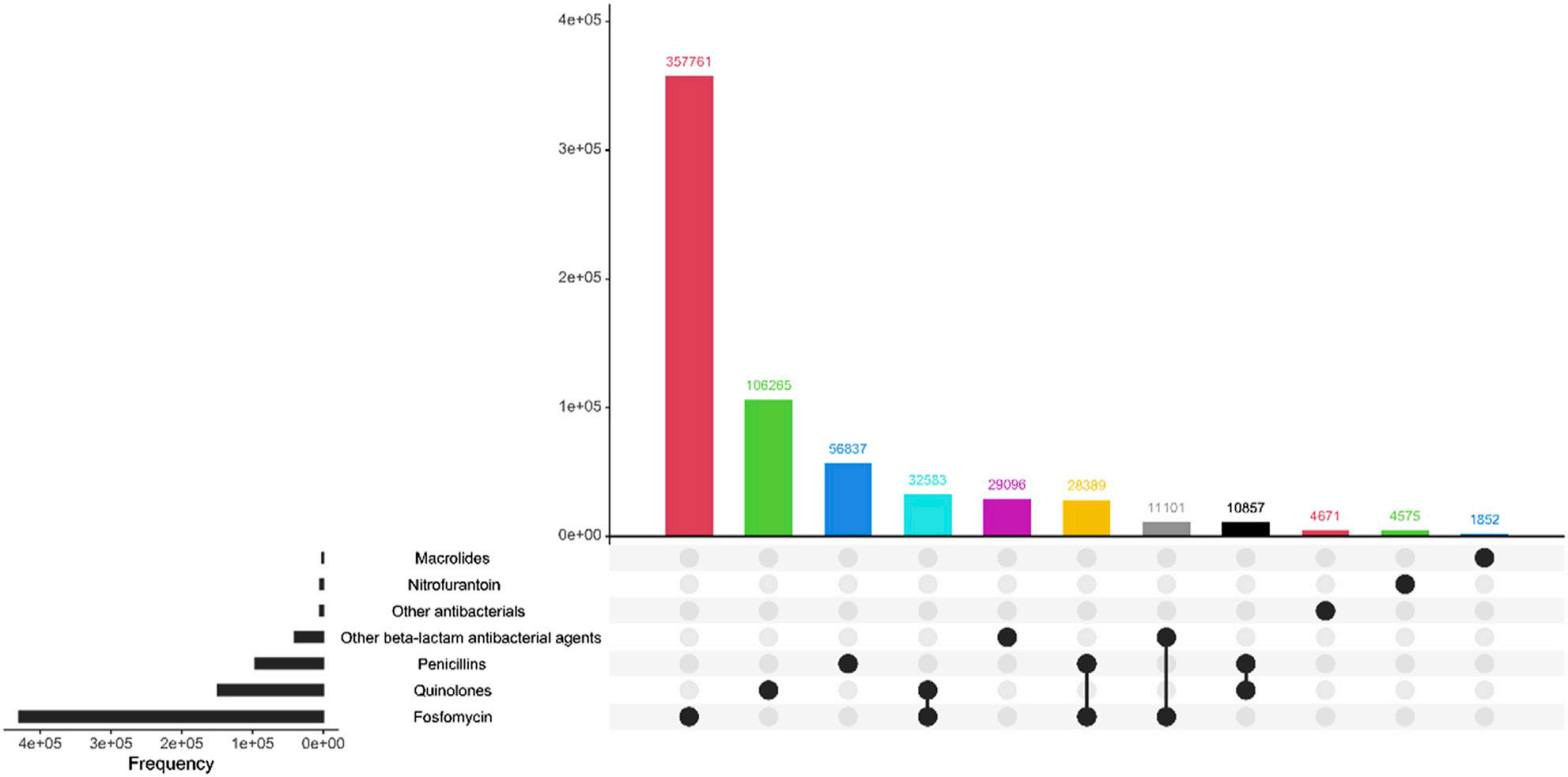
Antibiotic treatment of patients diagnosed with cystitis, by treatment group.

**FIGURE 4:**
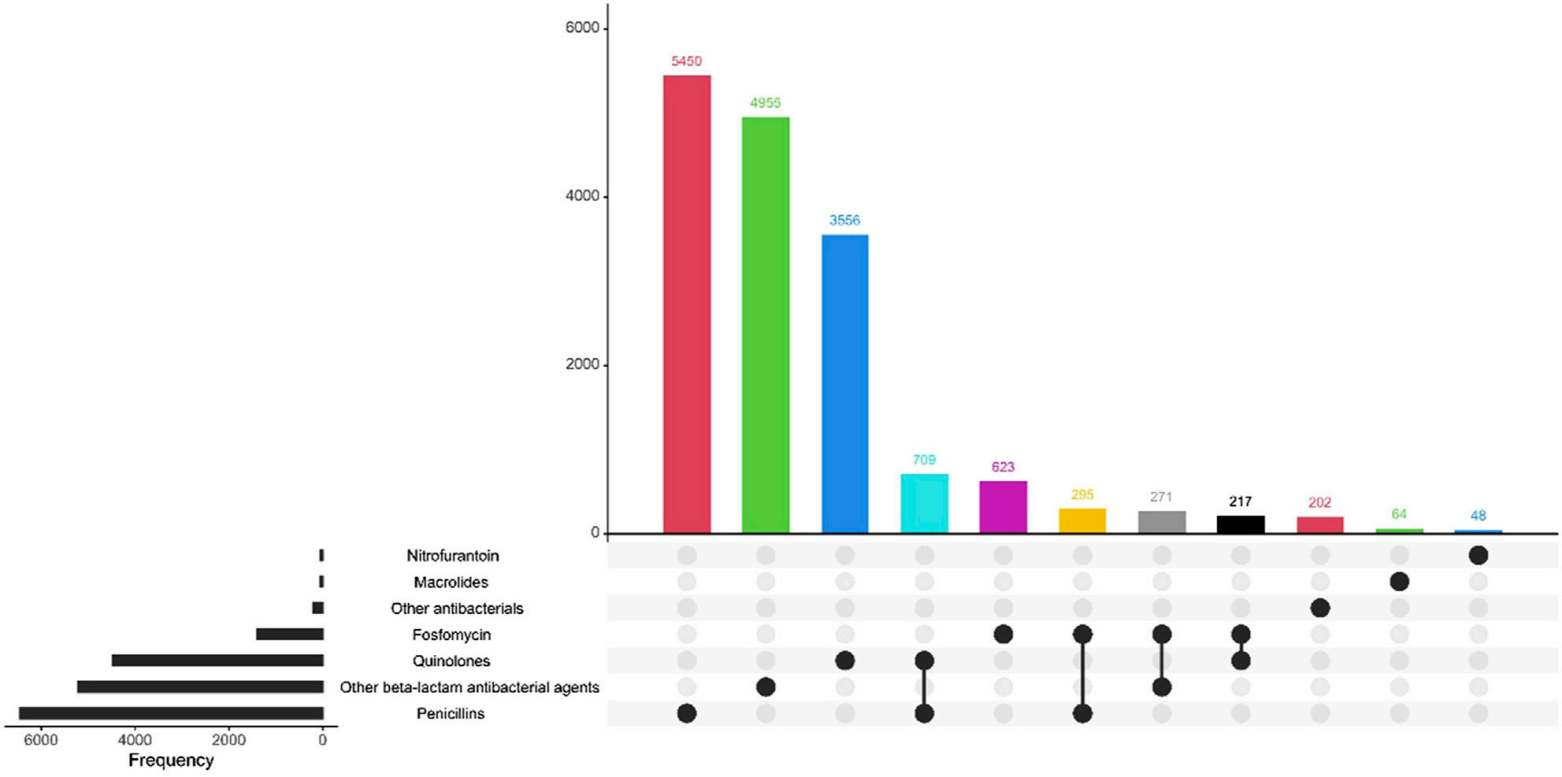
Antibiotic treatment of patients diagnosed with pyelonephritis, by treatment group.

The most prescribed antibiotic treatment in the cystitis group was fosfomycin, followed by treatment with quinolones. In contrast, in the pyelonephritis group, penicillins were the most frequently recorded treatment, followed by treatment with the other beta-lactam antibiotic group. 98.7% of the first-line antibiotics for uncomplicated UTI corresponded to fosfomycin. Table 4; Figures 2–4 describe the antibiotic treatments, including the most frequent drugs of the study.

**TABLE 4:** Antibiotic treatment of UTI.

Antibiotics	Cystitis	Pyelonephritis
Number of patients with antibiotic treatment	676,442	29,086
Treatment group (%)		
Quinolones^a^	106,265 (15.7)	3,556 (12.2)
Ciprofloxacin	39,913 (5.9)	2,707 (9.3)
Norfloxacin	62,337 (9.2)	587 (2.0)
Penicillins^a^	56,837 (8.4)	5,450 (18.7)
Amoxicillin	55,492 (8.2)	5,376 (18.5)
Fosfomycin	357,761 (52.9)	623 (2.1)
Macrolides^a^	1,852 (0.3)	64 (0.2)
Azithromycin	1,251 (0.2)	41 (0.1)
Nitrofurantoin	4,575 (0.7)	48 (0.2)
Other beta-lactam antibacterial agents^a^	29,096 (4.3)	4,955 (17.0)
Cefuroxime	22,732 (3.4)	3,123 (10.7)
Cefixime	5,460 (0.8)	1,360 (4.7)
Other antibacterials^a^	4,671 (0.7)	202 (0.7)
Trimethoprim + sulfamethoxazole	3,212 (0.5)	161 (0.6)
Quinolones + Penicillins	10,857 (1.6)	709 (2.4)
Quinolones + Fosfomycin	32,583 (4.8)	217 (0.7)
Penicillins + Fosfomycin	28,389 (4.2)	295 (1.0)
Fosfomycin + Other beta-lactam antibacterial agents	11,101 (1.6)	271 (0.9)
Other combinations	32,455 (4.8)	1,714 (5.9)

^a^ Only the most frequently registered combinations are listed.

Women who suffered from recurrent UTIs were older, and were more likely to be residents of nursing homes, as well as being more likely to report diabetes mellitus and dyslipidemia (Table 5). In terms of antibiotic dispensing, patients with recurrent UTIs have more antibiotics dispensed (in 3 or more dispensations) than patients with no record of recurrent UTI (Table 6). Fosfomycin was the most commonly reported treatment for recurrent UTI, reaching almost 70%, followed by quinolones, and penicillins.

**TABLE 5:** Sociodemographic and baseline clinical characteristics of patients diagnosed with recurrent UTI and no recurrent UTI.

Sociodemographic and baseline clinical characteristics	Recurrent	Non-recurrent	SMD^a^
Number of patients (%)	26,549 (3.7%)	698,048 (96.3%)	
Sociodemographic data			
Age			
Age (mean (SD))^b^	58.37 (23.04)	51.39 (20.47)	0.320
≤20	1,332 (5.0)	30,123 (4.3)	
(20,30]	3,086 (11.6)	100,745 (14.4)	
(30,40]	3,002 (11.3)	117,428 (16.8)	
(40,50]	2,901 (10.9)	112,039 (16.1)	
(50,60]	2,795 (10.5)	95,934 (13.7)	
(60,70]	3,261 (12.3)	89,079 (12.8)	
(70,80]	4,158 (15.7)	79,936 (11.5)	
>80	6,014 (22.7)	72,764 (10.4)	
Nursing home			
Nursing home (%)	3,670 (13.8)	33,242 (4.8)	0.316
Area			
Rural	3,931 (14.8)	107,673 (15.4)	0.129
Urban	17,220 (64.9)	482,785 (69.2)	
MEDEA Index (%)^c^			
Urban first quintile (least deprived)	3,069 (11.6)	87,888 (12.6)	0.096
Urban second quintile	3,210 (12.1)	95,381 (13.7)	
Urban third quintile	3,667 (13.8)	99,435 (14.2)	
Urban fourth quintile	3,697 (13.9)	101,536 (14.5)	
Urban fifth quintile (most deprived)	3,577 (13.5)	98,545 (14.1)	
Baseline clinical characteristics			
Smoking status (%)^d^			
Non-smoker	17,560 (70.6)	418,923 (65.8)	0.147
Former smoker	3,314 (13.3)	118,977 (18.7)	
Smoker	4,003 (16.1)	98,523 (15.5)	
Alcohol (%)^d^			
No risk	16,530 (77.3)	366,788 (70.8)	0.150
Moderate risk	4,759 (22.3)	148,813 (28.7)	
High risk	87 (0.4)	2,621 (0.5)	
Body Mass Index			
Body Mass Index (mean (SD))	27.72 (5.86)	27.77 (5.88)	0.008
Body Mass Index >30 (%)	4,952 (31.3)	115,601 (31.5)	0.004
Comorbidites no related to urinary tract			
Diabetes mellitus (%)	1,807 (6.8)	20,864 (3.0)	0.178
Dyslipidemia (%)	2,532 (9.5)	36,077 (5.2)	0.168
Diseases of the nervous system and Cerebrovascular diseases (%)	1,044 (3.9)	8,150 (1.2)	0.176
Musculoskeletal system and connective tissue diseases (%)	498 (1.9)	9,719 (1.4)	0.038
Diseases of the digestive system (%)	403 (1.5)	5,461 (0.8)	0.069
Comorbidites related to urinary tract			
Urinary litiasis (%)	230 (0.9)	2,840 (0.4)	0.058
Chronic renal insufficiency (%)	872 (3.3)	6,949 (1.0)	0.159

^a^ Standardized Mean Difference.

^b^ In years.

^c^ MEDEA, index was categorized in five quintiles.

^d^ Variables with missing data (8.7% in smoking status, 25.5% in alcohol, and 47.1% in body mass index).

**TABLE 6:** Treatment of recurrente UTIs and non-recurrent UTIs.

Treatments	Recurrent	Non-recurrent	SMD*
Number of patients	26,549 (3.7%)	698,048 (96.3%)	
Number of ATB in the first year after infection			
Mean (SD)	3.63 (2.61)	0.74 (1.30)	1.400
Median [IQR]	3.00 [2.00, 5.00]	0.00 [0.00, 1.00]	
0 packagings	1,652 (6.2)	414,125 (59.3)	1.926
1 packaging	2,704 (10.2)	165,092 (23.7)	
2 packagings	5,668 (21.3)	66,925 (9.6)	
3–5 packagings	11,741 (44.2)	44,086 (6.3)	
6–10 packagings	4,173 (15.7)	6,701 (1.0)	
>10 packagings	611 (2.3)	1,119 (0.2)	
Treatment group (%) and duration of treatment^b^			
Quinolones	13,284 (50.0)	72,261 (10.4)	0.959
Less 3 months	12,067 (90.8)	69,087 (95.6)	0.191
More than 3 months	1,217 (9.2)	3,174 (4.4)	
Penicillins	11,380 (42.9)	151,206 (21.7)	0.466
Less 3 months	10,453 (91.9)	144,061 (95.3)	0.140
More than 3 months	927 (8.1)	7,145 (4.7)	
Fosfomycin	18,497 (69.7)	82,855 (11.9)	1.454
Less 3 months	16,207 (87.6)	78,704 (95.0)	0.264
More than 3 months	2,290 (12.4)	4,151 (5.0)	
Nitrofurantoin	5,343 (0.8)	2,036 (7.7)	0.349
Less 3 months	4,659 (87.2)	1,760 (86.4)	0.022
More than 3 months	684 (12.8)	276 (13.6)	
Trimethoprim	5,079 (0.7)	1,846 (7.0)	0.328
Less 3 months	4,679 (92.1)	1,667 (90.3)	0.064
More than 3 months	400 (7.9)	179 (9.7)	
Macrolides	2,112 (8.0)	42,672 (6.1)	0.072
Less 3 months	2,008 (95.1)	41,057 (96.2)	0.056
More than 3 months	104 (4.9)	1,615 (3.8)	
Other beta lactam antibacterial agents	7,455 (28.1)	28,674 (4.1)	0.690
Less 3 months	6,798 (91.2)	27,323 (95.3)	0.164
More than 3 months	657 (8.8)	1,351 (4.7)	
Other antibacterials	15,811 (2.3)	2,550 (9.6)	0.314
Less 3 months	14,552 (92.0)	2,306 (90.4)	0.057
More than 3 months	1,259 (8.0)	244 (9.6)	
Other clinical events			
Urine culture (%)	8,152 (30.7)	186,146 (26.7)	0.089
Specialized care referrals (%)^c^	974 (3.7)	17,086 (2.4)	0.071

^a^ Standardized Mean Difference.

^b^ Duration of the first round of dispensations of this group during 1 year since index data. One patient could be in both groups.

^c^ Registration of specialized care referrals from primary care.

## Discussion

The study aimed to characterise UTIs over a 10-year period in Catalonia using the population-based database, describing the socio-demographic and clinical characteristics, as well as the management of the disease.

Regarding the age at which UTI was recorded in our database, it was similar for cystitis in other populations such as Switzerland (Plate et al., 2019), the Netherlands (Spek et al., 2020), and China (Wong et al., 2017) with ages ranging from 53 to 54 years. However, this was not the case for pyelonephritis, which in our setting was between 30 and 40 years of age, unlike other European country where younger ages were recorded (Isberg et al., 2019).

Diabetes is considered a risk factor for UTI (Bonkat et al., 2024), but it was not the first comorbidity recorded in our study. Nevertheless, the percentage of diabetes mellitus was similar to other countries, such as Sweden with 3.6% (Kornfält Isberg et al., 2019) but not in China, where it is higher (16.4%) (Wong et al., 2017). More women were diagnosed with dyslipidemia, although this is not considered a risk factor.

It should be noted that 17.8% of cystitis and 38.8% of pyelonephritis had no record of antibiotic treatment in the database, which is similar to other studies using databases (Isberg et al., 2019; Kornfält Isberg et al., 2021). However, studies with lower percentages around 7.6% can be found in the literature (Plate et al., 2020). This lack of record could be explained by the use of data from medical records, one of the limitations of observational studies. The percentage of antibiotic treatment recorded in our study was similar to other European studies conducted in Sweden (Kornfält Isberg et al., 2019; Kornfält Isberg et al., 2021), Germany (Gágyor et al., 2020); and Hong Kong (Wong et al., 2017). In the case of pyelonephritis, one of the possible reasons for the higher number of antibiotic non-registrations is referral to a hospital centre as a complicated UTI, as in-hospital antibiotic treatment was not available in our database.

In our setting, cystitis was most commonly treated with fosfomycin, in line with treatment guidelines, whereas pyelonephritis was treated with penicillins as a family of antibacterials. In a study published in 2017 involving a Spanish cohort, fosfomycin was already the most commonly prescribed antibiotic (used in almost 80% of cases), although the overall rate of antibiotic prescriptions related to UTI was higher (95.1%) (Butler et al., 2017).

One of the most significant findings of our study is the high percentage of fosfomycin used among Catalan prescribers. Two antibiotics are recommended by our national guidelines based on their proven efficacy, low rates of resistance, limited ecologial impact, and low propensity to select for resistance (Frimodt-Møller and Bjerrum, 2023; Llor et al., 2023; Bonkat et al., 2024), and our data suggest that healthcare professionals are adhering to these recommendations. Both antibiotics exhibit strong activity against *E. coli* and are considered first-line treatments in many countries due to their narrow spectrum and favorable safety profiles. However, the use of fosfomycin accounts for more than 98% of the prescriptions for these two recommended antibiotics, while nitrofurantoin is seldom used in our area. This limited use may be attributed to a warning issued in 2016 by the Spanish Agency of Drugs and Medicinal Products, advising against the use of nitrofurantoin for durations longer than 7 days (Martínez-Macías et al., 2018; Almirante Gragera et al., 2021). The use of these antibiotics for uncomplicated UTIs is also recommended in other European countries; however, prescribing patterns are more balanced elsewhere. In some countries, for example, nitrofurantoin is the most used antibiotic remaining the most prescribed agent in the Netherlands and parts of Scandinavia, while fosfomycin is favoured in countries like Germany (Malmros et al., 2019). Interestingly, a systematic review and meta-analysis evaluating the comparative efficacy and safety of fosfomycin and nitrofurantoin in the management of uncomplicated UTIs found a higher incidence of adverse events in the fosfomycin group compared to the nitrofurantoin group (Konwar et al., 2022). Nevertheless, nitrofurantoin is rarely prescribed locally, possibly because regional guidelines recommend it only as an alternative to fosfomycin (Almirante Gragera et al., 2021).

Urine culture was requested for approximately 30% of UTIs, which is in line with other European and Asian countries that report around 30%–40% (Plate et al., 2019; Koh et al., 2023). Due to the nature of the project, the reasons for the request were not available. It should be noted that a descriptive study of the adequacy of urine cultures in our setting has been carried out in one of the work packages of the ITUCAT project. It showed that the percentage of over-requesting urine cultures remains high, with one-fifth of cultures ordered for uncomplicated UTIs (Fernández-García et al., 2025). In addition, a very low percentage of sepsis was recorded, as in a study with database in England, with 0.5% sepsis registered (Gharbi et al., 2019).

In our study, 3.7% of the population reported recurrent UTI. This percentage is similar in different populations, ranging from 2.7% in an English population-based study to 6% in the Welsh population (Ahmed et al., 2019; Sanyaolu et al., 2024) although there are studies with higher percentages, such as a prospective study in Switzerland (Kornfält Isberg et al., 2021) or a study of women in the United States in which 30% of the women surveyed had recurrent UTIs (Gleicher et al., 2023).

The sociodemographic characteristics for recurrent UTIs are similar to other studies in the literature (Samimi et al., 2021; ten Doesschate et al., 2022; Gleicher et al., 2023; Sanyaolu et al., 2024), although the diagnosis of diabetes was reported at higher rates in the literature, reaching 22.1% (Sanyaolu et al., 2024). In studies conducted in Spain, a higher percentage of diagnosed diabetes mellitus and urolithiasis (Lorenzo-Gómez et al., 2015; Carrión-López et al., 2020) was observed than in our database.

Fosfomycin was the most frequently reported antibiotic treatment for recurrent UTIs during the yearly follow-up from the index date, with the lowest frequency in the 3-month period. This antibiotic is one of those used for prophylactic treatment according to clinical guidelines (Patología infecciosa en: Grupo de Trabajo de Enfermedades Infecciosas de la semFYC, 2017; Almirante Gragera et al., 2021; Bonkat et al., 2024). Furthermore, non-antibiotic measures for UTI prophylaxis are not included in the database, although there are several studies in the literature evaluating non-antibiotic treatments (Rui et al., 2022; Hayward et al., 2024; Heltveit-Olsen et al., 2024) to reduce antibiotic overuse and help prevent antimicrobial resistance.

One of the strengths of our study is the large sample size, which allowed us to obtain information from a large proportion of the female population in Catalonia. In addition, the information obtained corresponds to clinical practice in our primary care setting over a period of 10 years, which can be useful for observing the antibiotic stewardship in PHC, as shown in other studies in Europe (Mulder et al., 2019). Understanding how UTIs are treated enables us to determine whether clinical guidelines are being adhered to, ensure that patients are receiving appropriate treatment, and identify areas for improvement in disease management, if necessary, to help prevent antimicrobial resistance. On the other hand, the limitations of our study are those inherent to observational studies using data from electronic health records. Most importantly, causality cannot be established, and there may be bias due to confounding variables and inaccuracies in the records in the health registers. For instance, we acknowledge the potential for selection biases - patients with missing data may represent a healthier population–as well as possible errors arising from incomplete or inaccurate diagnostic records. Another limitation of the study is that our data focused on public primary care settings, so we do not have information from hospitals or private practices.

Our study provides information about the treatment and management of UTI in the women population in our setting, and this information is both important and useful for future research, such as the integration of hospital-based data, assessment of patient adherence to prescribed treatments, and evaluation of the long-term outcomes associated with various management strategies.

## Conclusion

We conducted a large-scale study of UTI in women in primary care. Almost the entire female population of Catalonia was included, allowing the description of women with a diagnosis of UTI and obtaining information of great importance for evaluating the management of UTI in our setting.

The sociodemographic characteristics are similar to other studies, both for UTIs and recurrent UTIs. In our study, the use of fosfomycin for the treatment of cystitis is the most commonly reported, in line with local guidelines. In contrast, antibiotic treatment for pyelonephritis does not follow local recommendations according to data from our study, which is based on primary care data. Evaluating the management and treatment of this infection helps us identify areas for improvement in primary care, with the aim of enhancing patient care and preventing antibiotic resistance.

## Data Availability

The datasets generated and/or analyzed during the current study are not publicly available due to patient privacy and data protection concerns, but they are available from the corresponding author on reasonable request.

## Abbreviations

ATC, Anatomical, therapeutic, chemical classification system; CMBD, Minimum Basic Data Sets; ICD, International Statistical Classification of Diseases; ICS, Institut Català de la Salut; SIDIAP, Information system for the development of research in Primary Care; UTIs, Urinary tract infections.
