# Supplementary material for: Urinary tract infections in women in Catalonia, Spain: a population-based observational cohort study in primary care
Source: Front Pharmacol. 2025 Sep 9;16:1593910. doi: 10.3389/fphar.2025.1593910 (PMC12455073; doi:10.3389/fphar.2025.1593910)
Supplement: Supplementary file 1 [file DataSheet1.pdf]

Supplementary material 1

Table 1. Clinical variables and health conditions with ICD-10-CM codes.

Disease of the genitourinary system		
Pyelonephritis	N10	Acute tubule-interstitial nephritis
Cystitis	N30	Cystitis
	N39	Other disorders of urinary system
Disease of the nervous system		
G20 - G26		Extrapyramidal and movement disorders
G30 - G32		Other degenerative diseases of the nervous system
G80 - G83		Cerebral palsy and other paralytic syndromes
Cerebrovascular diseases		
I60		Subarachnoid haemorrhage
I61		Intracerebral haemorrhage
I62		Other nontraumatic intracranial haemorrhage
I63		Cerebral infarction
I64		Stroke, not specified as haemorrhage or infarction
I65		Occlusion and stenosis of precerebral arteries, not resulting in cerebral infarction
I66		Occlusion and stenosis of cerebral arteries, not resulting in cerebral infarction
I67		Other cerebrovascular diseases
I68		Cerebrovascular disorders in diseases classified elsewhere
I69		Sequelae of cerebrovascular disease
Musculoskeletal system and connective tissue diseases		
M05 - M14		Inflammatory polyarthropathies
Diseases of the digestive system		
K50		Crohn disease
K51		Ulcerative colitis
K70 - K77		Diseases of liver

Table 2. Anatomical, therapeutic, chemical classification system (ATC) codes for drugs of interest.

Treatment group	ATC code included
Quinolones	J01MA
Penicillins	J01C
Fosfomycin	J01XX01
Macrolides	J01FA
Nitrofurantoin	J01XE01
Trimetropim	J01E
Other beta-lactam antibacterial agents	J01DB<br>J01DC<br>J01DD<br>J01DE
Other antibacterials	J01A<br>J01B<br>J01DF<br>J01DH<br>J01DI<br>J01FF<br>J01FG<br>J01G<br>J01MB<br>J01R<br>J01X
